# What Motivates Men to Improve Their Health? Understanding the Roles of Self-Esteem and Influential Others in Behaviour Change

**DOI:** 10.3390/nu16121916

**Published:** 2024-06-18

**Authors:** Lisa S. McNeill

**Affiliations:** Otago Business School, University of Otago, P.O. Box 56, Dunedin 9054, New Zealand; lisa.mcneill@otago.ac.nz; Tel.: +64-3-4795758

**Keywords:** self-esteem, male body image, health behaviours

## Abstract

The aim of this study was to examine men’s body image confidence, social reflectivity, body image perceptions and external information sources influence regarding body assessments. Data were collected via a cross-sectional survey and found that men have a low motivation toward physical health behaviour changes such as food, alcohol and exercise evaluation, and have generally positive views of their bodies overall. Relationship status, rather than age, defined behavioural and attitudinal differences within the men studied here. Men in this study were largely uninfluenced by celebrities or fashion in developing their own body image perceptions; single males were more likely to turn to friends, the female population generally and societal norms when evaluating themselves. Males in relationships however, weighted their partner’s opinion as the greatest influence, followed by their involvement in sport. This study offers an insight into the role of body confidence in male perspectives of the self, which is important for its intrinsic connection to motivations for health behaviours such as body weight management. This has implications for increasing the effectiveness of health-related product and service messaging, and public health messages regarding body weight management for men.

## 1. Introduction

Self-identity, the way in which one defines oneself, relative to others, has long been examined from the perspective of women. As traditional notions of gender in self-image blur, men have begun to receive attention from researchers concerned with consumption behaviour as an extension of the self [1,2]. Much of this focus has, however, centred on products embraced by male consumers in a subversion of traditionally female categories of consumption (such as fashion and cosmetics). Where gaps still lie is in understanding men’s perceptions of themselves, and how notions of self-identity might motivate men toward image management behaviours, including physical health-related behavioural change. There is evidence that men’s body image satisfaction is influenced strongly by spouses and similar familial relationships, however. This study addresses those gaps by examining the impact of relationship status on men’s body image perceptions and body confidence, as well as the impact of personal, social and external influence sources in body maintenance behaviours.

Now that physical health behaviours are increasingly recognised as identity related, research that examines the relationship between body image, identity and lifestyle is useful in that it focuses on the notion that positive self-perception is fundamentally linked to an individual’s satisfaction with their own body image [3,4,5]. Where prior research has linked negative self-perceptions of women to motivations for physical change, little work has examined men’s perceptions of their bodies in relation to self-perception, or motivations for men to engage in physical health-change behaviours. This study examines the notion of male body image satisfaction, the extent to which one is happy with one’s body, as it relates to motivation toward identity management behaviours, such as body modification through diet and exercise.

### 1.1. Self Esteem and Body Satisfaction

Satisfaction with one’s body is said to be driven by several social factors, including media, society, friends and family, with individuals utilising social comparison theory to compare their body to that of those around them [6]. Regarding health, there is conflicting evidence surrounding the role of body image associations and lifestyles of healthy or unhealthy behaviour. For example, some studies find that positive self-esteem and body image beliefs generally encourage more positive fitness and health-related behaviours (in college age students of both sexes) [7]. Others find that a high body dissatisfaction (in adult women) is associated with stronger fitness and health motivations toward appearance and weight [8]. Some recent research suggests that body dissatisfaction (in adolescents of both sexes) is associated with negative body management behaviour, including unhealthy weight loss strategies, lower physical activity, dysfunctional exercise, and increased computer usage [9]. While prior research has examined women, children and teenagers/adolescents in respect of their body image and self-esteem, little work has examined the adult male (beyond contrasting them to women). Further, there are gaps in understanding differences in body image perceptions and self-esteem between adult male subjects.

### 1.2. Male Body Image and Weight

Where perceptions of body image relate to health management actions, prior research confirms that weight evaluation plays a critical part in the relationship between body image and physical activity [9]. Gillison et al. [10] found that negative body weight perceptions endorsed extrinsic motivations for physical activity (e.g., exercise for weight loss), thus negatively predicting self-determined motivations for exercise behaviour. However, evaluation of physical activity is often absent from research that examines body image [9]. Those studies that do examine physical activity alongside body image report inconsistences between samples. For example, some studies show that participation in physical activity by adolescent girls is not associated with body image satisfaction [11]. Others claim that exercise helps to improve body image in adolescents of all sexes [12].

One explanation for many of the difference observed when examining the impact of physical activity on body image is that body image satisfaction in these contexts is driven by a self-evaluation of body weight, which then raises questions as to the relative accuracy of self-assessments of the body and the resultant satisfaction with the self, between individuals. Regarding males, recent studies in developed countries show that boys and men are more likely to underestimate their body weight [9,13] than women are. However, a larger proportion of men than women are seen as overweight (based on BMI) [13]. Those who overestimate body weight are deemed more likely to engage in weight management behaviours (such as reducing intake of dairy, fats, and oils), yet, male adolescents, who are more likely to underestimate their weight than females, are also more likely to consume higher volumes of foods associated with weight gain [9].

### 1.3. Lifestyle and Male Health Behaviours

The psychological quality of life cannot be separated from physical quality regarding body image [14], thus recent research calls for further study of the relationship between individual lifestyle factors and body image [13]. There is a growing body of research that examines body image and lifestyle influences in childhood and adolescent body maintenance motivation, yet gaps remain for work that focuses on adults [15]. Further, the exact links between body image and many individual lifestyle factors (beyond age) are still to be determined. Of those studies that have focused on adult lifestyles, many have considered body image as a female concern, adding to the misunderstanding of outcomes of male body image perceptions that tends to dominate such work [16]. It is accepted, however, that body image is a multifactorial construct that is affected by a group of psychological, physiological and social factors, each with different levels of influence on health behaviours at different lifestyle stages [13].

### 1.4. Relationships and Body Image Satisfaction

Self-reporting happiness studies indicate that those with strong connections to others are happier than those without. In addition, particular interpersonal relationships, such as a romantic attachment, account for the greatest difference in satisfaction with the self [17,18]. If the singular lifestyle factor of relationship status is then examined further, it has been found that relationship status relates strongly to health-related quality of life perceptions, with those in relationships less worried about their health overall. Further, men in relationships appear to be the least worried of all groups, with single men and women more likely to be occupied with food control as a health mechanism related to body image perceptions [3].

Some research suggests that men in romantic relationships are said to be happier overall than women in these partnerships, with males more likely to benefit from the emotional support and gratification provided by a partner than vice versa [17]. When the influence of these interpersonal relationships on body image satisfaction is considered, it appears that peer reflexivity impacts the body weight assessments (and subsequent body satisfaction) of women but not of men [19], suggesting a difference in the role of social forces on male assessments of their own body. Indeed, other research suggests that women are more likely than their male counterparts to correctly evaluate their own weight in relation to the general population [20] and that married men are more likely to be ‘happily’ obese than their never married or previously married counterparts [21], i.e., acknowledging being overweight but not feeling negative or motivated toward change, regarding this. Thus, following calls for further study of adults, men and individual lifestyle factors, this study examines relationship status in adult males, as related to body image satisfaction, by proposing the following:

**H1.**  *That males in relationships have more positive body esteem than do single males*;

and

**H2.**  *That males in relationships are likely to have more positive body appearance perceptions than do single males.*

Social identity theory notes that self-evaluative or self-conceptual outcomes rely heavily on social context, irrespective of the cognitive categorisation of attributes (in terms of the recognition of normative beliefs or behaviours) [22]. Where body image dissatisfaction in women occurs, it is often in relation to comparison to a standard of social beauty perceived to be above that of the consumer’s own [23,24]. Further, females tend to be influenced more by outside factors, such as the media and society more generally, leading to an assessment of body image related to one’s ‘value’ in the social world [4,24]. In contrast, male body image satisfaction, when influenced by others, is said to be evaluated based on the opinions of close friends (in the case of young men) and spouses or family members (in the case of older males) [25].

One could propose that males in relationships are more likely to assess their body image based on the opinions of those closest to them, and less likely to self-categorise based on external sources of body image perceptions. Adding further emphasis to the proposed body satisfaction of men in relationships are findings such as those which state that, after the end of a relationship, males are likely to revise any weight gain and maintain this weight loss (where any female weight loss in the same instance tends to be short-lived) [21]. These findings suggest that, irrespective of actual weight or appearance, men in relationships are generally happy with their body, when compared to women, and, when single, are prompted to revise their body assessment (evidenced by the weight loss seen in newly separated males in the Sobal et al. [15] study). Thus:

**H3.**  *That males in relationships are more likely to emphasise personal over social body image perception antecedents than are single males;*

and

**H4.**  *That males in relationships are less likely to be impacted by external influence sources than are single males.*

## 2. Materials and Methods

### 2.1. Survey and Scale Items

The research utilised a quantitative approach and an online survey (hosted via Qualtrics) to collect data. This study used a market research company (Research First) to access respondents, from a total database of 1 million adult contacts. Male, NZ members of the research panel were e-mailed the link to the survey by Research First and asked to complete qualifying questions on their age and sexual orientation. Respondents were recruited in a quota format in four banded age groups (18–24 years; 25–34 years; 35–44 years and 45–65 years). Respondents in this study were limited to heterosexual males, to allow for between-participant comparison, in relation to questions regarding sexual attractiveness and the influence of women, and of romantic partners, on body image perceptions, thus reducing the potential for multiple moderating variable influences. It is reasonable to propose that body image perceptions and goals may differ between sexual orientations, hence the decision to limit the focus of this study to a single sexual orientation. Survey questions examined self-perceptions of the participant’s bodies, the influence of others on these perceptions and overall evaluations of personal physical appearance in relation to the body. Participants were also asked a series of behavioural health-related questions, examining participants’ purposeful moderation of food, alcohol, fat, snacks/treats and increased exercise or participation in sport.

The scales employed were drawn or developed from existing studies of body and appearance perceptions [24,25,26]. Personal body image views, as per H1, were examined using a version of Rosenberg’s [25] self-esteem scale, modified to the body context (“myself” in the original scale replaced with “my body”). The reliability of this scale has been tested since its inception, with some psychologists criticising its one-dimensional nature [27]. In this study, the scale yielded a Cronbach’s alpha of 0.595, which is not ideal. Some prior studies have yielded an alpha coefficient of 0.86 using the scale, indicating good internal reliability; however, these studies have largely been undertaken with small samples (less than one hundred participants) [28]. Nevertheless, the Rosenberg scale has been accepted as an appropriate measure of self-esteem over the past 55 years.

To counter the low alpha of the Rosenberg self–esteem scale, and to examine H2, a shortened version of Netemeyer et al.’s [26] vanity scale was employed, with the ‘Physical-Concern’ and ‘Physical-View’ item sets used as a single scale to measure appearance perceptions. The Physical-Concern item set comprises five reflexive, self-determined appearance perceptions (e.g., ‘the way I look is extremely important to me’) and the Physical-View item set comprises six response-oriented appearance perceptions (e.g., “my looks are very appealing to others”). In this study, the shortened Netemeyer et al.’s [26] scale yielded a Cronbach’s alpha of 0.914.

The last two scales employed were developed for this study from McNeill and Firman’s [25] qualitative study of ideal body image factors for men. The first of the scales developed for this study, examining H3, consisted of twelve items that examined significant male body image perception factors, including muscularity; leanness; masculinity; age; fitness goals; sexual attractiveness; body satisfaction; friend opinions; family opinions; health; lifestyle; stomach fat; and sporting ability (Cronbach’s alpha: 0.891). The second scale developed for this study, concerned with H4, examined external and internal influence factors on men’s personal body image perceptions, with ten items, including celebrity influence; family; friends; women (generally); romantic partners; society; fashion; age; health; and sport (Cronbach’s alpha: 0.889).

Participants were asked to rate survey items on a seven-point Likert scales as follows: 1 = strongly agree/extremely important; 2 = agree/important; 3 = somewhat agree/somewhat important; 4 = neither agree or disagree/neither important nor unimportant; 5 = somewhat disagree/somewhat unimportant; 6 = disagree/unimportant; and 7 = strongly disagree/not important at all. As the data were collected, responses were checked for completion time and central tendency, with concerning responses excluded. The survey contained two attention check questions, as well as some reverse items, which allowed for further data checking.

### 2.2. Sample

A total of 355 complete responses were collected. Respondent ages were roughly equally split between four age bands (18–24; 25–34; 35–44; 45–65 years) and with the ethnicity of participants representing proportionally the major ethnic groups of New Zealand (53.1% Caucasian). A total of 69.9% of respondents had a secondary school-level qualification or higher. A total of 31.3% of respondents participated in exercise at least weekly, 48.9% monthly or less and 19.8% did not exercise at all. A total of 67.8% of respondents were in a romantic relationship and 32.2% were single. Regarding relationship status and age, 18–24-year-old participants were the least likely to be in a relationship (48% of this group), while approximately one-third of each of the other three age groups of participants were in relationships.

### 2.3. Analysis

Survey responses were exported to IBM Statistical Package for Social Sciences (SPSS) 29 for analysis. To validate the measures, a factor analysis was run on the scales. All scales had Kaiser–Meyer–Olkin (KMO) values of above the threshold of 0.6 and the Eigenvalues indicated there was one dominant factor in all scales. The principal factor analyses further confirmed that all values loaded onto one component, and as such, the scales can be seen to be reliable. Results were obtained by comparing means and via ANOVA.

## 3. Results

### 3.1. Male Health-Related Behaviours

Of the age bands represented, participation in sport was relatively equally represented across all age bands, with no indication that one age group had a significantly greater or lesser participation overall. When age was examined as an initial predictor of body image attitudes (per scale), no significant differences in mean responses between age groups were found. Regarding health-related behaviours, participants did not exhibit a strong motivation toward health behavioural changes, with reductions in food and alcohol or increases in sport and exercise relatively moderate across the group (Table 1). The only significant difference in health-related behaviour was seen in relation to increasing sport participation, where single males were less likely to attempt to increase their sports participation than were those in relationships.

### 3.2. Men’s Body Assessments and Self-Esteem

All the men in this study were generally positive about their own body but remained neutral in response to statements indicating extreme negativity, rather than disagreeing outright with statements such as “I am inclined to think that I am a failure in relation to my body” or “I feel I do not have much to be proud of in relation to my body”. However, where significant differences in the group means were seen, males in relationships were more positive than single males overall, with an emphasis on affirmative comparison of their body to others and satisfaction with their body generally (refer to Table 2). This supports H1.

### 3.3. Male Body Confidence

All the men in this study were moderately confident about their own body and remained neutral in response to statements indicating extreme body confidence, rather than disagreeing outright (such as “people are envious of my good looks” and “I have the type of body that people want to look at”). Where significant differences were seen between groups, males in relationships again exhibited greater confidence than did single males, rating their looks and sexual attractiveness higher. This supports H2. Single males, however, were more likely to see a need for a continued effort in looking their best and an awareness of how others might perceive their appearance, indicating the potential for greater reflexivity in their assessments of self (refer Table 3).

### 3.4. Male Body Perceptions–Influences

Both single males and those in a relationship indicated their health as the most influential factor on how they thought about their body, and celebrities and fashion as least important in influencing their body image. Where differences between the groups were significant, single males were more likely to be influenced by their friends, by women and by society generally, than were males in relationships. Males who were part of a couple tended to be influenced more by their partner, and by their enjoyment of sports, than did single males (refer Table 4). These findings give support to H3 and H4.

### 3.5. Physical Appearance Factors–Key Indicators

Single males weighted most motivating factors as more important than did their non-single counterparts, and where this difference was significant, single males in the study were more likely to emphasise socially reflective factors, such as being lean, what friends think of their appearance and having a visibly flat stomach, than were males in relationships (refer to Table 5). This supports H3 and H4. Men in relationships in this study emphasised the importance of being healthy, having a good lifestyle balance and being able to participate in sports when compared to single participants, but the difference was not significant. Both groups indicated health, lifestyle and body satisfaction as the most important factors in motivating their overall body image perceptions.

## 4. Discussion

For health-related products and services and public health messages regarding body weight management to be effective, research that explores differentiating factors within specific gender groups is required. The aim of this study was to examine how men’s body image confidence and perceptions, along with their social reflectivity, influenced their personal body assessments. The association between physical health and body image perceptions has been established in prior research, particularly among adult populations. Further, prior research confirms the intrinsic connections between body image, self-esteem and motivations for health behaviours such as body weight management [3]. Those who are more preoccupied with their body not fitting a perceived ideal are more likely to be motivated towards change regarding physical exercise or dieting [3]. However, the benefits of this association are reliant on critically accurate body assessments—essentially, that those who need to modify their lifestyle or health behaviours recognise the need for change in their own body [9]. The differences apparent in how individuals evaluate their body, and how this motivates body-related health behaviours such as diet and exercise, highlight a need for research that examines specific demographic and sociographic groups. While previous research has examined women, children and teenagers/adolescents in respect of their body image and self-esteem, little work has examined the adult male (beyond contrasting them to women). Further, there are gaps in understanding differences in body image perceptions and self-esteem between adult male subjects. This study contributes to our understanding of the heterosexual adult male and their body image evaluations and motivations toward body health-related behaviour.

The prior literature has indicated a relationship between body image positivity and motivation toward exercise in adolescents, but studies of adults indicate that body satisfaction and health-promoting lifestyles are not strongly related [13]. This study finds that adult males are generally very positive about their bodies, with satisfaction overall in how their body compares to others. This was particularly evident amongst adult males in romantic relationships, who rated themselves as better looking and more sexually attractive than did single men. In the literature concerning adolescent perceptions of the body, body image concerns are not said to promote healthy behaviours; rather, lower body esteem promotes unhealthy behaviour regarding nutrition and exercise avoidance. In contrast, the single males in this study, who exhibited lower body confidence than those males in relationships, were more motivated toward a continued effort in looking their best (based on an awareness of how others might perceive their physical appearance). In this study, age was not a significant predictor of body image perceptions or health behaviours.

Low body dissatisfaction is known to have a significant impact on lifestyle behaviours, particularly those related to fitness and health motivations [3] However, while female perceptions of their body and related body image concerns have long been a subject of research interest, consideration of males has largely been limited to contrast between them and women, regarding women’s proposed body negativity and unhealthy behaviour [9]. The male participants of this study, despite representing four separate age cohorts from early adulthood to 65 years, did not indicate significant motivations toward health-directed body management behaviours, including monitoring their fat and alcohol intake, moderating food intake for health or increasing their exercise. This is a useful finding, as it illustrates a widespread issue in male health campaigning—the lack of strong motivation for change across all ages when considering easy-to-implement physical health actions. The current research suggests that social reflexivity may be a better motivator for behaviour change amongst these men, with key external influences for both single men and men in relationships identified. Further, having a positive body image may be linked to more sustainable, long-term health-promoting behaviours rather than drastic, short-term changes such as dieting or extreme exercise programs. Limitations of this study include the single country sample (New Zealand) and the cross-sectional nature of the data collection. A study that includes physical health data by participant, collected over a period, would add weight to our understanding of the research questions proposed here. Further, where relationships have been the focus of this study, it is recommended that future research extends further than heterosexual males. Other recommendations for future research would include the examination of other lifestyle factors, to better understand the relative weight of influence of the single/relationship construct. The poor reliability of the Rosenberg self-esteem scale indicates the need for further exploration of this construct, potentially via the development of a new scale, in future research.

## 5. Conclusions

Public health promotion messages and health-related products and services targeting men are often focused on the issue of body weight management. This study indicates the lack of a common set of drivers toward body maintenance and weight management across all males, but it does indicate different foci for males in relationships compared to those who are single. Age was not found to be a significant influence for male views of the body; rather, body image satisfaction tended to be influenced by other aspects of life satisfaction, as indicated by relationship status. This is an important and useful finding for those developing health/weight messages targeting men in New Zealand. Health promotion that is effective for each of the two cohorts examined here should activate key motivators of body image satisfaction in any messaging, such as the view of friends (for single males) and partners (for those in relationships), rather than focusing on personal health management drivers, including negative assessments of the self.

## Figures and Tables

**Table 1:** Health-related behaviour.

On the Scale Below, Indicate How Regularly You Engage in the Following Activities?	Do You Have a Partner or Significant Other					
	Yes (n = 240)		No (n = 355)		Total (n = 355)	
	Mean	SD	Mean	SD	Mean	SD
Watch the amount of fat I consume	3.65	1.583	3.64	1.585	3.65	1.581
Reduce my alcohol intake	3.24	1.736	3.22	1.964	3.23	1.810
Moderate my food intake	3.52	1.360	3.55	1.416	3.53	1.376
Cut back on snacks and treats	3.47	1.477	3.50	1.495	3.48	1.481
Increase my exercise	3.39	1.374	3.52	1.489	3.43	1.411
* Attempt to increase my participation in sport	3.77	1.698	4.44	1.846	3.99	1.773

* Significant at 0.05, 1 = always; 7 = never.

**Table 2:** Men’s body esteem.

To What Extent Do You Agree with the Following Statements about Your Body?	Do You Have a Partner or Significant Other						*p*-Value
	Yes (n = 240)		No (n = 115)		Total (n = 355)		
	Mean	SD	Mean	SD	Mean	SD	
On the whole, I am satisfied with my body	3.34	1.325	3.86	1.567	3.51	1.427	0.001 *
At times, I think my body is no good at all	4.02	1.574	3.87	1.565	3.97	1.570	0.410
I feel that my body has a number of good attributes	2.87	1.010	3.10	1.068	2.94	1.033	0.051
I am able to do things as well as most other people	2.75	1.103	3.02	1.256	2.84	1.160	0.042 *
I feel I do not have much to be proud of in relation to my body	4.29	1.477	4.14	1.388	4.24	1.449	0.354
I certainly feel useless at times	4.49	1.664	4.07	1.599	4.35	1.653	0.024 *
I feel that I am at least on an equal plane with others	3.23	1.136	3.52	1.180	3.32	1.157	0.026 *
I wish I could have more respect for my body	3.73	1.513	3.61	1.336	3.69	1.457	0.451
I am inclined to feel that I am a failure in relation to my body	4.65	1.598	4.17	1.574	4.50	1.604	0.009 *
I take a positive attitude towards my body	3.16	1.171	3.39	1.219	3.23	1.190	0.084

* significant at 0.05, 1 = strongly agree; 7 = strongly disagree.

**Table 3:** Appearance perceptions.

To What Extent Do You Agree with the Following Statements about Yourself?	Do You Have a Partner or Significant Other						*p*-Value
	Yes (n = 240)		No (n = 115)		Total (n = 355)		
	Mean	SD	Mean	SD	Mean	SD	
The way I look is extremely important to me	3.37	1.437	3.16	1.328	3.30	1.405	0.187
I am very concerned about my appearance	3.59	1.441	3.32	1.386	3.50	1.427	0.101
* I would feel embarrassed if I was around people and did not look my best	3.97	1.510	3.50	1.471	3.82	1.511	0.006 *
* Looking my best is worth the effort	3.43	1.327	3.12	1.278	3.33	1.318	0.037 *
It is important that I always look good	3.68	1.372	3.53	1.410	3.63	1.384	0.331
People notice how attractive I am	3.99	1.366	4.07	1.497	4.02	1.408	0.626
* My looks are very appealing to others	3.93	1.295	4.34	1.450	4.06	1.359	0.008 *
* People are envious of my good looks	4.40	1.488	4.77	1.358	4.52	1.456	0.022 *
* I am a very good looking individual	3.89	1.360	4.40	1.407	4.05	1.394	0.001 *
* My body is sexually appealing	3.98	1.466	4.43	1.475	4.13	1.482	0.008 *
* I have the type of body that people want to look at	4.29	1.413	4.63	1.416	4.40	1.421	0.031 *

* significant at 0.05, 1 = strongly agree; 7 = strongly disagree.

**Table 4:** Importance of influential factors on male body image.

How Important Are the Following in Influencing the Way You Think about Your Own Body?	Do You Have a Partner or Significant Other						*p*-Value
	Yes (n = 240)		No (n = 115)		Total (n = 355)		
	Mean	SD	Mean	SD	Mean	SD	
Celebrities	5.25	1.542	5.04	1.613	5.18	1.566	0.255
Family	3.63	1.522	3.55	1.540	3.60	1.527	0.639
* Friends	3.70	1.487	3.17	1.213	3.52	1.425	0.001 *
* Women	3.55	1.497	3.09	1.472	3.40	1.503	0.006 *
* Your partner/loved one	2.54	1.206	3.25	1.611	2.77	1.389	0.000 *
* Society, generally	3.86	1.482	3.45	1.384	3.73	1.462	0.014 *
Fashions	4.45	1.560	4.22	1.497	4.38	1.542	0.176
Age	3.73	1.500	3.50	1.360	3.66	1.458	0.174
Health	2.80	1.367	2.84	1.225	2.81	1.321	0.772
* Sport	3.62	1.523	4.06	1.693	3.76	1.591	0.015 *

* significant at 0.05, 1 = extremely important; 7 = not important at all.

**Table 5:** Key indicators of desired physical appearance.

How Important Is the Following to You?	Do You Have a Partner or Significant Other						*p*-Value
	Yes (n = 240)		No (n = 115)		Total (n = 355)		
	Mean	SD	Mean	SD	Mean	SD	
Being muscular	3.76	1.344	3.70	1.364	3.74	1.349	0.725
* Being lean	3.40	1.247	3.00	1.068	3.27	1.206	0.003 *
Being seen as very masculine	3.93	1.427	3.82	1.302	3.90	1.387	0.462
Looking better than other males in my age group	3.95	1.447	3.77	1.465	3.89	1.454	0.252
Reaching my personal fitness goals	3.31	1.390	3.28	1.335	3.30	1.370	0.826
Attracting a partner/being attractive to a partner	3.03	1.229	2.90	1.370	2.99	1.276	0.389
Feeling satisfied with my body	2.78	1.065	2.65	.983	2.74	1.040	0.282
* What my friends think of my physical appearance	3.85	1.419	3.50	1.280	3.73	1.383	0.025 *
What my family thinks of my physical appearance	3.82	1.444	3.77	1.366	3.81	1.418	0.711
Being healthy, rather than just looking good	2.49	1.153	2.54	.985	2.50	1.101	0.680
Having a good lifestyle balance	2.47	1.035	2.57	1.101	2.50	1.056	0.390
* Being in the right physical shape for the sports I enjoy	3.05	1.345	3.36	1.488	3.15	1.398	0.056 *
* Having a flat stomach	3.36	1.273	3.06	1.223	3.26	1.263	0.038 *

* Significant at 0.05, 1 = extremely important; 7 = not important at all.

## Data Availability

Data are unavailable due to privacy or ethical restrictions.
